# Augmentation Stability of Guided Bone Regeneration for Peri-Implant Dehiscence Defects with L-shaped Porcine-Derived Block Bone Substitute

**DOI:** 10.3390/ma14216580

**Published:** 2021-11-02

**Authors:** Jae-Hong Lee, Eun-Hee Jung, Seong-Nyum Jeong

**Affiliations:** Department of Periodontology, Daejeon Dental Hospital, Institute of Wonkwang Dental Research, Wonkwang University College of Dentistry, Daejeon 35233, Korea; joyus94@naver.com (E.-H.J.); seongnyum@wku.ac.kr (S.-N.J.)

**Keywords:** bone regeneration, bone substitute, cone-beam computed tomography, dental implants, wound healing

## Abstract

Block bone substitutes have better augmentation stability for guided bone regeneration (GBR) than particulate bone substitutes. This study sought to determine whether GBR with an L-shaped porcine block bone (DPBM-C) differs from GBR with an L-shaped bovine block bone (DBBM-C) based on clinical, radiographic, and volumetric outcomes for peri-implant dehiscence defects. A total of 42 peri-implant defects were grafted with 20 L-shaped DPBM-C and 22 DBBM-C groups. The horizontal and vertical thicknesses of the augmented hard tissue were measured using sagittal cone-beam computed tomography, and the volumetric tissue change was evaluated by stereolithography image superimposition. Postoperative discomfort, early wound healing outcomes, and implant stability were also assessed. Among the clinical (subjective pain and swelling, wound dehiscence, membrane exposure, and periotest values), radiographic (changes in horizontal and vertical hard tissue thickness), and volumetric parameters of the L-shaped DPBM-C and DBBM-C groups during the healing period, only the periotest values showed a statistically significant difference (0.67 ± 1.19, *p* = 0.042). Within the limitations of this study, an L-shaped DPBM-C is not inferior to an L-shaped DBBM-C based on their clinical, radiographic, and volumetric outcomes for GBR of peri-implant dehiscence defects.

## 1. Introduction

Guided bone regeneration (GBR) is a clinically predictable and effective technique for augmenting horizontal and vertical alveolar bone defects around dental implants [1,2,3]. One systematic review demonstrated that the clinical outcomes of GBR in peri-implant dehiscence defects had survival rates of 92.6–100% [2]. Another systematic review reported that implants simultaneously placed with GBR, regardless of the type of barrier membrane and grafting material, show an overall survival rate of 95.7% (range, 84.7–100%) [3]. A more recent retrospective study confirmed that implant surgery with simultaneous GBR had a 97.95% survival rate (implant present in the oral cavity independent of complications) and a success rate (being free of mechanical and/or biological complications over the entire functional period) of approximately 90%, similar to that of non-grafted implants. [1].

Various bone grafting biomaterials are currently being developed for GBR applications [3,4]. Autogenous bone is used as a “gold standard” due to its osteogenic, osteoinductive, and osteoconductive properties; however, its high resorption rate, donor site morbidity, and the limited amount of bone that can be harvested are considered as major disadvantages [5]. As an alternative, allografts, xenografts, and alloplastic bone materials are effectively applied in clinical practice; there is still controversy about which of them is superior [6].

The block bone substitutes showed better space-making and maintenance capacity than the particulate bone substitutes, which is one of the prerequisites for predictable bone regeneration and successful long-term outcomes in GBR [7,8,9]. Among the xenogeneic block bone substitutes, deproteinized bovine bone mineral with a 10% collagen (DBBM-C) block bone substitute, which was predominantly used for alveolar ridge preservation, provides good maneuverability and favorable adaptations to the horizontal and vertical bone defect [10,11,12].

A recent in vitro study demonstrated that DBBM-C, appropriately trimmed to an L-shape, resisted the pressure of the overlying mucoperiosteal flap and significantly improved the hard tissue stability of the augmented site during the suture procedure [13]. On the other hand, research evaluating the clinical efficacy of GBR with an L-shaped DBBM-C for peri-implant dehiscence defects is relatively scarce [14]. In addition, to the best of our knowledge, no study has evaluated the usefulness and clinical applicability of an L-shaped deproteinized porcine bone mineral with 10% collagen (DPBM-C).

Therefore, our study aimed to determine if GBR with an L-shaped DPBM-C with a resorbable collagen membrane differs from GBR with an L-shaped DBBM-C based on the clinical, radiographic, and volumetric outcomes for peri-implant dehiscence defects.

## 2. Materials and Methods

### 2.1. Ethical Statements

This retrospective study adhered to the principles of the Helsinki Declaration of 1975, as revised in 2013, and was approved by the Institutional Review Board of Daejeon Dental Hospital, Wonkwang University (approval No. W2105/002-001) [15]. The study also followed the STROBE guidelines for the conduct and reporting of observational studies [16].

### 2.2. Participants

The clinical, radiographic, and volumetric data were retrospectively retrieved from the electronic dental records of 42 patients who underwent GBR with L-shaped soft block bone substitutes between September 2016 and May 2021 at the Daejeon Dental Hospital, Wonkwang University, Daejeon, Korea.

Inclusion criteria:

- Presence of peri-implant buccal dehiscence defects (≥1 mm);
- Re-entry surgery within 4–6 months;
- Healthy or mild systemic diseases
  (American Society of Anesthesiologists physical status classification I/II);
- Good or acceptable oral hygiene
  (full-mouth bleeding score on probing and full-mouth plaque score < 25%).

Exclusion criteria:

- Implant surgery with GBR within 1 month after tooth extraction;
- Heavy smokers (≥10 cigarettes/day);
- Uncontrolled diabetes mellitus or bone metabolic diseases.

### 2.3. Surgical Procedure

**Figure 1:**
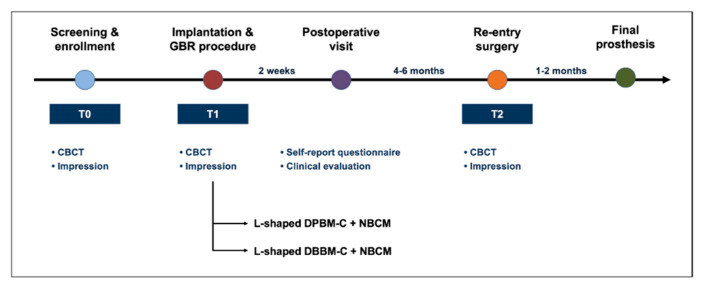
Schematic presentation of the treatment protocol and time points. CBCT sagittal cone-beam computed tomography; DBBM-C, demineralized bovine bone mineral with 10% collagen; DPBM-C, demineralized porcine bone mineral with 10% collagen; GBR, guided bone regeneration; NBCM, absorbable native bilayer collagen membrane.

**Figure 2:**
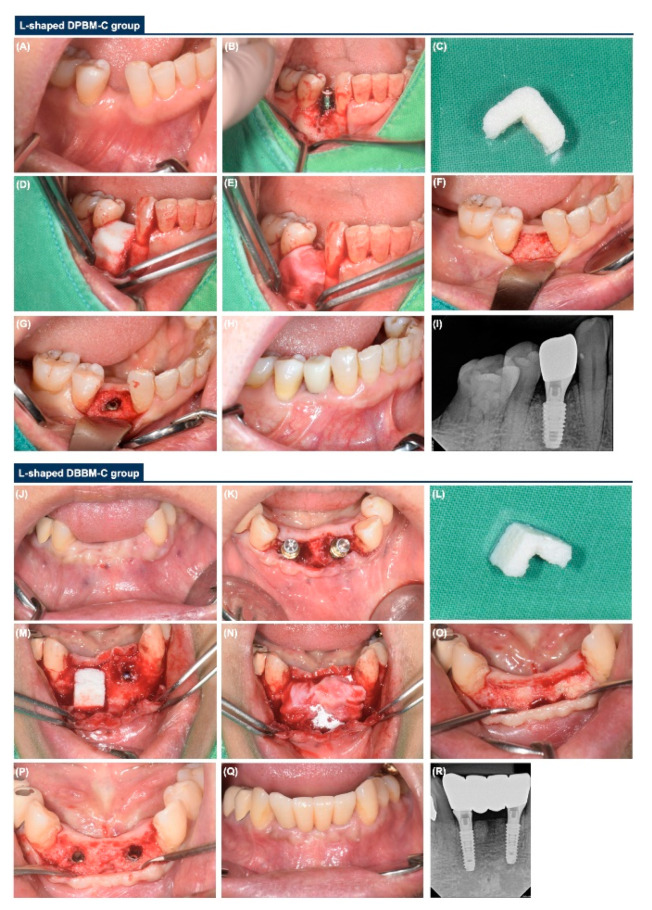
Representative clinical and radiographic images of the L-shaped DPBM-C and DBBM-C groups. (**A**) Facial view before implant surgery. (**B**) Peri-implant dehiscence defect before GBR. (**C**) DPBM-C trimmed to an L-shape. (**D**,**E**) GBR with an L-shaped DPBM-C and NBCM. (**F**,**G**) Facial view during re-entry surgery. (**H**,**I**) Clinical and radiographic view at final prosthesis delivery. (**J**) Facial view before implant surgery. (**K**) Occlusal view during implant surgery. (**L**) DBBM-C trimmed to an L-shape. (**M**,**N**) GBR with an L-shaped DBBM-C and NBCM. (**O**,**P**) Facial view during the re-entry surgery. (**Q**,**R**) Clinical and radiographic view at final prosthesis delivery.

All implant surgeries with GBR were conducted by a board-certified periodontist (JHL) (Figure 1). Full-thickness flaps were elevated, and vertical releasing incisions were made when necessary. A sandblasted large-grit acid-etched surface implant fixture (Osstem TSIII^®^, Osstem, Seoul, Korea; Superline^®^, Dentium, Seoul, Korea; SLActive^®^ BLT, Institut Straumann AG, Basel, Switzerland) was placed according to the manufacturer’s instructions (Figure 2).

### 2.4. Re-Entry Sugery

Re-entry surgery was conducted between 4 and 6 months, and full-thickness flaps were elevated under local anesthesia. After manual screw tightening and the confirmation of the implant-healing abutment complex, the periotest values (PTVs) were measured using the Periotest device (Periotest M^®^, Medizintechnik Gulden, Modautal, Germany). Primary flap closure was performed using interrupted sutures with 4–0 e-PTFE.

- L-shaped DPBM-C group: DPBM-C (Legograft^®^, Purgo Biologics, Seongnam, Korea), which was composed of a porcine-derived bone mineral matrix from cancellous bone and 10% atelocollagen from porcine tendon, was directly trimmed to an L-shape using a #15 blade. DPBM-C was manually adapted to the peri-implant dehiscence defect without using additional fixation devices (e.g., bone screws, pins, bone tack, or titanium mesh), and the defect was augmented to ≥1 mm of the buccal and occlusal aspects. The peri-implant dehiscence defect was augmented to ≥1 mm of the buccal and occlusal aspects. The L-shaped DPBM-C was covered with an absorbable native bilayer collagen membrane (NBCM, Geistlich Bio-Gide^®^, Geistlich Pharma AG, Wolhusen, Switzerland).
- L-shaped DBBM-C group: DBBM-C (Geistlich Bio-Oss^®^ Collagen, Geistlich Pharma AG, Wolhusen, Switzerland) was appropriately trimmed to an L-shape and applied to the peri-implant dehiscence defect. DBBM-C was manually adapted to the peri-implant dehiscence defect without using additional fixation devices, and the defect was augmented to ≥1 mm of the buccal and occlusal aspects. The defects were augmented to achieve a ≥1 mm over-contour for both the buccal and occlusal aspects. Subsequently, the L-shaped DBBM-C was covered with the NBCM.

For tension-free closure, a gentle and sufficient periosteal releasing incision was made on the underside of the mucoperiosteal flap using a #15 blade. Then, primary flap closure was achieved using interrupted and horizontal mattress sutures with 4–0 e-PTFE (Biotex^®^, Purgo, Seongnam, Korea) and 6–0 nylon sutures (Monosyn, B. Braun, Aesculap, Center Valley, PA, USA). All patients were instructed about oral hygiene techniques at home and received medications (including amoxicillin 500 mg and ibuprofen 200 mg, tid for 5–7 days) and a mouthwash (0.12% chlorhexidine, bid for 2 weeks).

### 2.5. Radiograhic Analysis

**Figure 3:**
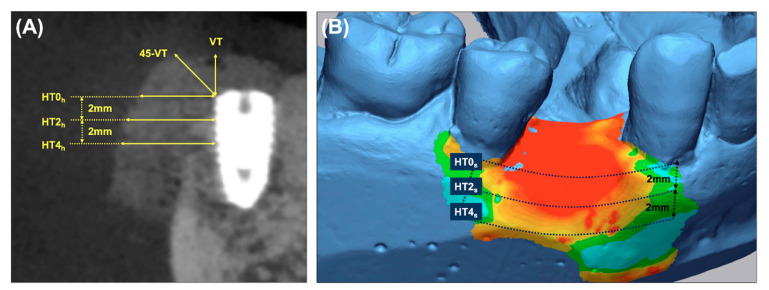
Schematic diagram of radiographic and volumetric outcome measurement. (**A**) Measure the horizontal and vertical thickness of the augmented hard tissue using sagittal CBCT images. HT0h, 2h, and 4h: Horizontal thicknesses at 0, 2, and 4 mm apically below the implant fixture shoulder; VT and 45-VT: Vertical thickness following the long axis of the fixture shoulder and positive 45° relative to the long axis of the fixture shoulder. (**B**) The horizontal thickness of the augmented soft tissue determined using stereolithography. HT0s, 2s, and 4s: the region of interest was limited by the mid-point of the facial cementoenamel junction of the mesial and distal teeth and extended 0, 2, and 4 mm apically below the buccal crest.

All patients underwent cone-beam computed tomography (CBCT, CS 8100 3D^®^, Carestream, Rochester, New York, NY, USA) examinations before the implant surgery (screening and enrollment, T0), immediately after implant surgery with GBR (T1), and on re-entry surgery (T2). Anonymized digital imaging and communication in medicine CBCT images were collected, and radiographic measurements were performed by one calibrated examiner (EHJ) using 3D imaging software (OnDemand 3D^®^ version 1.0.10.7510, Cybermed, Seoul, Korea). Intra-examiner reliability was estimated using the intra-class correlation coefficient (mean 0.85, range, 0.72–0.98) (Figure 3A).

### 2.6. Volumetric Analysis

The region of interest (ROI) encompassing the implant surgery with the GBR site was extra-orally scanned using a digital scanner (Trios3^®^, 3Shape, Copenhagen, Denmark) at T0, T1, and T2. To measure the soft-tissue contour alterations, the two sets of stereolithography (STL) files (T0–T1 and T0–T2) were superimposed by one examiner (EHJ) using the best-fit alignment method and image analysis software (Geomagic Control X version 2018, 3D Systems, Rock Hill, SC, USA) (Figure 3B).

### 2.7. Self-Reported Questionnaire and Clinical Analysis

Postoperative discomfort (including severity and duration of subjective pain and swelling) was assessed using a visual analog scale (VAS) (0–10; 0 = no pain and swelling, 10 = worst pain and swelling) 2 weeks after implant surgery [17]. Wound dehiscence and membrane exposure were also evaluated by one examiner (JHL).

### 2.8. Outcome Variables

- Primary outcome: To measure the horizontal thickness of the augmented hard tissue, lines perpendicular to the long axis of the fixture at its shoulder (HT0h) and 2 mm (HT2h) and 4 mm (HT4h) below it were drawn on the sagittal CBCT images.
- Secondary outcomes: (a) The vertical thickness (VT) of the augmented hard tissue following the long axis of the implant and the 45° vertical thickness (45-VT) at a 45° positive angle relative to the long axis of the fixture were measured on the sagittal CBCT images. (b) The horizontal thickness of the augmented volume was measured on the cross-sectional images of the STL products. The ROI was limited by the mid-point of the facial cementoenamel junction of the mesial and distal teeth and extended 4 mm apically. At the cross-section of the baseline, the lines parallel to the occlusal plane were drawn at the buccal crest (HT0s) and 2 mm (HT2s) and 4 mm (HT4s) below it. (c) Subjective postoperative discomfort and the early wound healing outcomes were assessed using a self-report questionnaire and based on the clinical evaluation during the suture removal. (d) The stability of the implants (PTVs) were measured during the re-entry surgery.

### 2.9. Statistical Analysis

The sample size was calculated based on a two-sided significance level of 0.05 and sufficient power (>80%) to detect the inter-group differences between the primary outcomes using G*Power software (version 3.1.9.4, Franz Faul, ChristianAlbrechts-Universität Kiel, Kiel, Germany) [18]. The results are expressed as frequencies (*n*), proportions (%), mean, standard deviation (SD), median, and first and third quartiles. The Shapiro–Wilk test was used to test for data normality, and the chi-squared test, Fisher’s exact test, and Student’s t-test were used to determine the significance of differences between the two groups. All calculations were performed using MedCalc (version 19.1.3, Mariakerke, Belgium), and *p* values of <0.05 denoted statistical significance.

## 3. Results

### 3.1. Baseline Characteristics

A total of 42 patients comprising 19 males (45.2%) and 23 females (54.8%) with a mean age of 58.9 years (range, 24–77 years) were eligible for retrospective evaluation (maxillary incisor, *n* = 14 (33.3%); maxillary posterior, *n* = 7 (16.7%); mandibular anterior, *n* = 14 (33.3%); mandibular posterior, *n* = 7 (16.7%)). The relevant baseline characteristics of the enrolled patients are shown in Table 1.

### 3.2. Radiographic and Volumetric Outcomes

**Figure 4:**
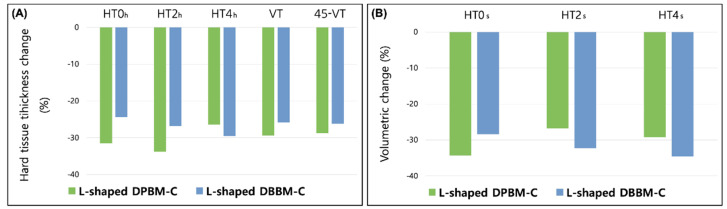
Comparison of radiographic and volumetric outcomes of the L-shaped DPBM-C and DBBM-C groups. (**A**) Horizontal and vertical changes in the mean percentage of hard tissue thickness during the healing period (T1 and T2) measured at HT0h, HT2h, HT4h, VT, and 45-VT. (**B**) Horizontal changes in the mean percentage of soft tissue thickness during T1 and T2 were measured at HT0s, HT2s, and HT4s. No significant differences between the compared two groups were found (*p* > 0.05).

Changes in the radiographic and volumetric outcomes of the augmented site after GBR treatment of the peri-implant dehiscence defects after a mean duration of the healing period of 5.4 months are listed in Table 2. The changes in the horizontal and vertical thicknesses of the hard tissue in the L-shaped DPBM-C were −0.83 ± 0.74 mm (−31.5%) at HT0h, −0.82 ± 0.79 mm (−33.8%) at HT2h, −0.61 ± 0.56 mm (−26.4%) at HT4h, −0.65 ± 0.68 mm (−29.4%) at VT, and −0.70 ± 0.71 mm (−28.8%) at 45-VT. The corresponding changes in the L-shaped DBBM-C were −0.63 ± 0.59 mm (−24.4%) at HT0h, −0.63 ± 0.73 mm (−26.8%) at HT2h, −0.74 ± 0.49 mm (−29.5%) at HT4h, −0.77 ± 0.90 mm (−25.8%) at VT, and −0.77 ± 1.03 mm (−26.2%) at 45-VT, respectively (Figure 4A). There were no significant differences between the two groups at T1 and T2.

Volumetric changes in the L-shaped DPBM-C were −1.42 ± 1.09 mm (−34.3%) at HT0s, −1.54 ± 1.20 mm (−36.8%) at HT2s, and −1.27 ± 1.07 mm (−29.2%) at HT4s. The corresponding changes in the L-shaped DBBM-C were −1.24 ± 0.97 mm (−28.4%) at HT0s, −1.46 ± 1.02 mm (−32.3%) at HT2s, and −1.50 ± 0.96 mm (−34.6%) at HT4s, respectively (Figure 4B). There were also no significant differences between the two groups at T1 and T2.

### 3.3. Postoperative Discomfort and Wound Healing Outcomes

The severities of subjective pain and swelling (*p* = 0.501, 0.385, respectively) and their durations (*p* = 0.569, 0.086, respectively) were not significantly different in the L-shaped DPBM-C and DBBM-C groups. The early wound healing outcomes, including wound dehiscence and membrane exposure, were also not significantly different between the two groups (*p* = 0.348). Detailed information on postoperative discomfort and the wound healing outcomes is provided in Table 3.

### 3.4. Stability of the Dental Implants

**Figure 5:**
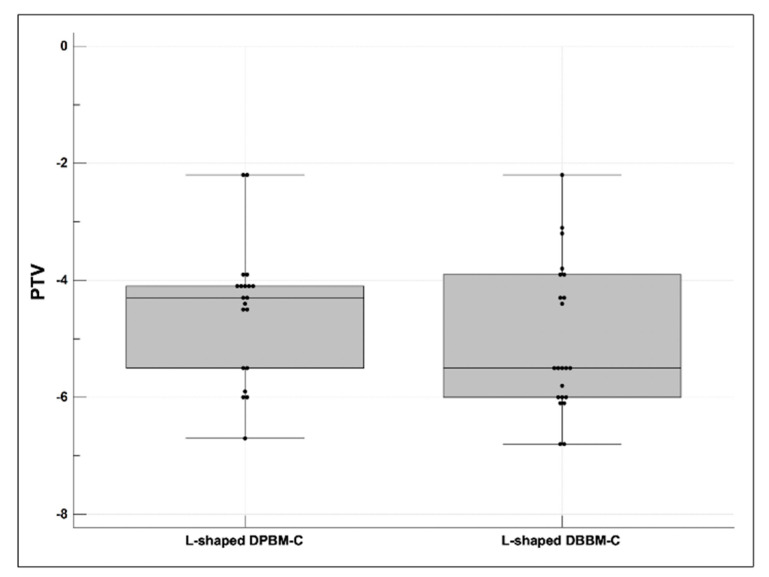
Comparison of the periotest values (PTVs) after re-entry surgery. The PTVs were expressed using box and whisker plots, and they were insignificantly different in the compared groups (*p* > 0.05).

The mean PTVs showed statistically significant differences between the L-shaped DPBM-C (−4.52 ± 1.16, median −4.30 (first and third quartiles −5.50; −4.10)) and DBBM-C (−5.19 ± 1.22, median −5.50 (first and third quartiles −6.00; −4.15)) groups (*p* < 0.05). The box and whisker plots are shown in Figure 5.

## 4. Discussion

Several in vitro and human studies have reported that block bone substitutes have a better hard tissue dimensional stability of GBR than particulate bone substitutes [7,8,9]. One in vitro CBCT study reported that the reduction in block bone substitutes was 2.4 ± 9.2% at HT0, 28.0 ± 11.9% at VT, and 24.8 ± 10.2% at 45-VT, which was statistically significantly lower than the reduction in particulate bone substitutes (*p* < 0.05) [7]. Another recent randomized controlled clinical study by Benic et al. reported that block bone substitutes (mean 22.5%) were associated with lower horizontal hard tissue reductions than particulate bone substitutes (mean 81.8%) on average in GBR for peri-implant dehiscence defects (*p* < 0.001) [8]. A recent systematic review evaluating the clinical efficacy of bone grafting materials also confirmed that the horizontal (mean 4.5 ± 1.2 mm) and vertical (mean 5.8 ± 2.8 mm) augmentations of block bone substitutes were significantly superior and more stable than the horizontal (mean 3.7 ± 1.2 mm) and vertical (mean 3.7 ± 1.4 mm) augmentations of particulate bone substitutes (*p* < 0.05) [9].

A recently devised soft block bone substitute, especially the L-shaped DBBM-C, was associated with a significantly improved augmentation stability of further peri-implant dehiscence defects [13]. An in vitro study reported that GBR with an L-shaped DBBM-C (HT0: −2.4 ± 9.2%, VT: −28.0 ± 11.9%, and 45-VT: −24.8 ± 10.2%) was associated with a lower horizontal hard tissue reduction than GBR with DBBM during primary wound closure (*p* < 0.05), which is consistent with the results of our study [13]. A recent clinical study also reported that the changes in the L-shaped DBBM-C were −0.63 ± 0.55 mm (−19.5%) at HT0, −0.77 ± 0.60 mm (25.5%) at VT, and −0.74 ± 0.54 (23.0%) mm at 45-VT; the corresponding changes in the DBBM were −1.30 ± 0.77 mm (40.3%), −1.57 ± 0.67 mm (52.0%), and −1.47 ± 0.69 mm (45.9%), respectively; statistically significant differences between the two groups were found (*p* < 0.05) [14].

Several animal and clinical trials have demonstrated that DPBM is not inferior to DBBM based on clinical and histological outcomes [19,20,21,22]. Furthermore, DPBM carries no potential risk of transmitting prion diseases [19]. Bae et al. compared the histological outcomes of DPBM and DBBM in rat calvarial defects and found no significant differences in the new bone area and volume, osteogenic ability, or gene expression between the two groups [20]. Comparative randomized clinical trials of DPBM and DBBM for alveolar ridge preservation of compromised and damaged extraction sockets found no significant differences in the hard and soft tissue dimensions and histologic variables (proportions of residual biomaterial, newly formed bone, and non-mineralized tissue) [21,22]. Another recent long-term clinical study also supported the finding that DPBM had a similar biocompatibility to that of DBBM [23,24].

Previous data suggest that the L-shaped DBBM-C (−0.74 ± 0.54 mm, −23.0%) showed a significantly better augmentation stability than the I-shaped DBBM-C (−1.43 ± 0.52 mm, −38.5%) at maintaining the occluso-buccal space, which is represented by 45-VT (*p* < 0.05) [14]. Urban et al. insisted on the importance of achieving space in the occluso-buccal corner, because the pressure caused by the flap may displace the bone substitutes placed in the coronal aspect [25]. This study is an extension of our research in which an L-shaped soft block bone substitute has advantages in resisting pressure and maintaining the occluso-buccal space.

This study evaluated the differences between the L-shaped DPBM-C and DBBM-C groups. Among the clinical (subjective postoperative discomfort, early wound healing outcomes, and implant stability), radiographic (changes in vertical and horizontal hard tissue thickness), and volumetric (changes in horizontal soft tissue thickness) parameters evaluated for the comparison between the two groups, only PTV showed a statistically significant difference (difference 0.67 ± 1.19, 95% confidence interval 0.02–1.32, *p* = 0.042); however, it has a minor clinical significance on implant stability. Based on our findings and on those of previous clinical, radiological, and histomorphological studies, an L-shaped DPBM-C, similar to an L-shaped DBBM-C, has good biocompatibility and is expected to be potentially useful in clinical practice, especially in GBR for peri-implant dehiscence defects.

The present study has several limitations. First, due to its retrospective design, the difference in numbers between the two groups was not adjusted and varied. Although this study focused on contained and buccal dehiscence intrabony defects, the treatment modality based on the selection of either DPBM-C and DBBM-C was not clear in clinical settings. In addition, preoperative soft and hard tissue parameters (e.g., gingival thickness, amount of keratinized gingiva, and periodontal pocketing or esthetics on the adjacent teeth) are important factors influencing the outcome of the GBR procedure, but this was not evaluated in the current study. Therefore, all clinical, radiographic, and volumetric outcomes were carefully interpreted, from which conclusions were drawn. The heterogeneity of the enrolled patients within each time point for tooth extraction was another limitation of this study. Further organized, prospective randomized clinical trials are required to confirm the efficacy of an L-shaped DPBM-C in clinical practice.

## 5. Conclusions

Within the limitations of a retrospective study, GBR with an L-shaped soft block bone substitute is a favorable treatment modality for peri-implant dehiscence defects. The L-shaped DPBM-C was not inferior to the L-shaped DBBM-C based on the clinical, radiographic, and volumetric outcomes during the healing period.

## Figures and Tables

**Table 1:** Baseline characteristics of the enrolled patients in the L-shaped DPBM-C and DBBM-C groups.

Variables	L-shaped DPBM-C (*n* = 20)	L-shaped DBBM-C (*n* = 22)	Total (*n* = 42)	*p* Value
Sex				
Male	11 (55.0%)	8 (36.4%)	19 (45.2%)	0.231
Female	9 (45.0%)	14 (63.6%)	23 (54.8%)	
Age				
Mean ± SD	58.9 ± 7.7	58.0 ± 13.6	58.4 ± 11.1	0.797
Smoking habits				
Non-smoker	12 (60.0%)	13 (59.1%)	25 (59.5%)	0.756
Former smoker	3 (15.0%)	5 (22.7%)	8 (19.0%)	
Smoker (<10 cigarettes/day)	5 (25.0%)	4 (18.2%)	9 (21.4%)	
Diabetes mellitus				
Yes	6 (30.0%)	5 (22.7%)	11 (26.2%)	0.596
Location				
Maxillary anterior	6 (30.0%)	8 (36.4%)	14 (33.3%)	0.923
Maxillary posterior	3 (15.0%)	4 (18.2%)	7 (16.7%)	
Mandibular anterior	7 (35.0%)	7 (31.8%)	14 (33.3%)	
Mandibular posterior	4 (20.0%)	3 (13.6%)	7 (16.7%)	

**Table 2:** Changes in hard and soft tissue thickness outcomes of the augmented site after GBR treatment of peri-implant dehiscence defects.

	L-shaped DPBM-C (*n* = 20)			L-shaped DBBM-C (*n* = 22)		
	T0–T1	T0–T2	*p* Value	T0–T1	T0–T2	*p* Value
Changes in hard tissue thickness (mm)						
HT0_h_	2.46 ± 1.06 (2.60, (1.60, 3.25))	1.63 ± 0.87 (1.42, (0.92, 2.20))	<0.001	2.50 ± 0.85 (2.33, (2.09, 2.98))	1.88 ± 0.85 (1.99, (1.21, 2.12))	<0.001
HT2_h_	2.19 ± 0.90 (2.20, (1.28, 2.85))	1.37 ± 0.75 (1.25, (0.70, 2.09))	<0.001	2.35 ± 0.85 (2.38, (1.85, 2.90))	1.71 ± 0.86 (2.01, (0.98, 2.34))	<0.001
HT4_h_	2.21 ± 0.87 (2.20, (1.29, 2.85))	1.60 ± 0.74 (1.54, (1.04, 2.12))	<0.001	2.58 ± 1.03 (2.61, (1.85, 3.01))	1.84 ± 0.94 (1.73, (1.12, 2.23))	<0.001
VT	2.28 ± 0.89 (2.38, (1.60, 2.95))	1.62 ± 0.91 (1.88, (0.69, 2.30))	<0.001	2.57 ± 0.89 (2.20, (2.01, 3.01))	1.79 ± 0.70 (1.77, (1.47, 2.04))	<0.001
45-VT	2.34 ± 1.01 (2.30, (1.41, 2.95))	1.64 ± 0.97 (1.48, (0.72, 2.64))	<0.001	2.57 ± 0.75 (2.53, (2.09, 2.98))	1.80 ± 0.76 (1.77, (1.19, 2.04))	<0.001
Changes in volume (mm)						
HT0_s_	3.97 ± 0.94 (4.12, (3.31, 4.50))	2.55 ± 0.98 (2.33, (2.03, 3.28))	<0.001	4.22 ± 0.86 (4.21, (3.44, 4.63))	2.98 ± 0.91 (3.05, (2.30, 3.47))	<0.001
HT2_s_	4.03 ± 0.99 (4.12, (3.34, 4.50))	2.49 ± 1.11 (2.33, (1.83, 3.35))	<0.001	4.32 ± 1.01 (4.28, (3.85, 5.02))	2.86 ± 0.97 (2.93, (2.33, 3.52))	<0.001
HT4_s_	4.17 ± 1.10 (4.17, (3.23, 4.90))	2.90 ± 1.11 (2.89, (2.15, 3.41))	<0.001	4.28 ± 0.86 (4.17, (3.75, 4.83))	2.78 ± 0.97 (2.93, (2.20, 3.14))	<0.001

Data are expressed as mean ± standard deviation (median, first and third quartiles). *p* values for comparisons between T0 and T1 and T0 and T2.

**Table 3:** Postoperative discomfort and early wound healing outcomes.

Variables	L-shaped DPBM-C (*n* = 20)	L-shaped DBBM-C (*n* = 22)	*p* Value
Subjective pain			
Severity (VAS) ^a^	4.8 ± 1.5	4.5 ± 1.6	0.501
Duration (days)	4.3 ± 2.5	4.9 ± 2.3	0.569
Subjective swelling			
Severity (VAS) ^a^	4.7 ± 2.0	4.2 ± 1.5	0.385
Duration (days)	6.9 ± 2.5	5.5 ± 2.5	0.086
Wound dehiscence and membrane exposure			
No	17 (85.0%)	20 (90.9%)	0.348
Yes	3 (15.0%)	2 (9.1%)	

Abbreviation: VAS, visual analog scale. Data are expressed as mean ± standard deviation. ^a^ Severity was assessed using the VAS score (0 = no pain and swelling, 10 = worst pain and swelling).

## Data Availability

Data available on request from the authors.
